# 2-[(*E*)-(5-Amino-2,3-diphenyl­quinoxalin-6-yl)imino­meth­yl]-4-bromo­phenol

**DOI:** 10.1107/S1600536808017716

**Published:** 2008-06-13

**Authors:** Hoong-Kun Fun, Reza Kia, Paul R. Raithby

**Affiliations:** a X-ray Crystallography Unit, School of Physics, Universiti Sains Malaysia, 11800 USM, Penang, Malaysia; b Chemistry Department, University of Bath, Claverton Down, Bath BA2 7AY, England

## Abstract

The title compound, C_27_H_19_BrN_4_O, is a mono-anil Schiff base ligand. Three intra­molecular O—H⋯N and N—H⋯N hydrogen bonds involving the hydr­oxy and amino groups generate *S*(6) and *S*(5) ring motifs, respectively. In the crystal structure, weak inter­molecular N—H⋯O and C—H⋯O hydrogen bonds together with π–π inter­actions [centroid–centroid distances = 3.628 (3)–3.729 (3) Å] link neighboring mol­ecules.

## Related literature

For details of hydrogen-bond motifs, see: Bernstein *et al.* (1995 ▶). For bond-length data, see: Allen *et al.* (1987 ▶). For related structures see, for example: Corden *et al.* (1996 ▶); Govindasamy *et al.* (1999 ▶). For applications and bioactivities see, for example: Blower (1998 ▶); Cohen & Schmidt (1964 ▶); Granovski *et al.* (1993 ▶); Kia *et al.* (2004 ▶); Li & Chang (1991 ▶); Shahrokhian *et al.* (2000 ▶); Uhlenbrock *et al.* (1996 ▶); Unaleroglu & Hokelek (2002 ▶). For related literature, see: Anderson *et al.* (1997 ▶); Blower (1998 ▶).
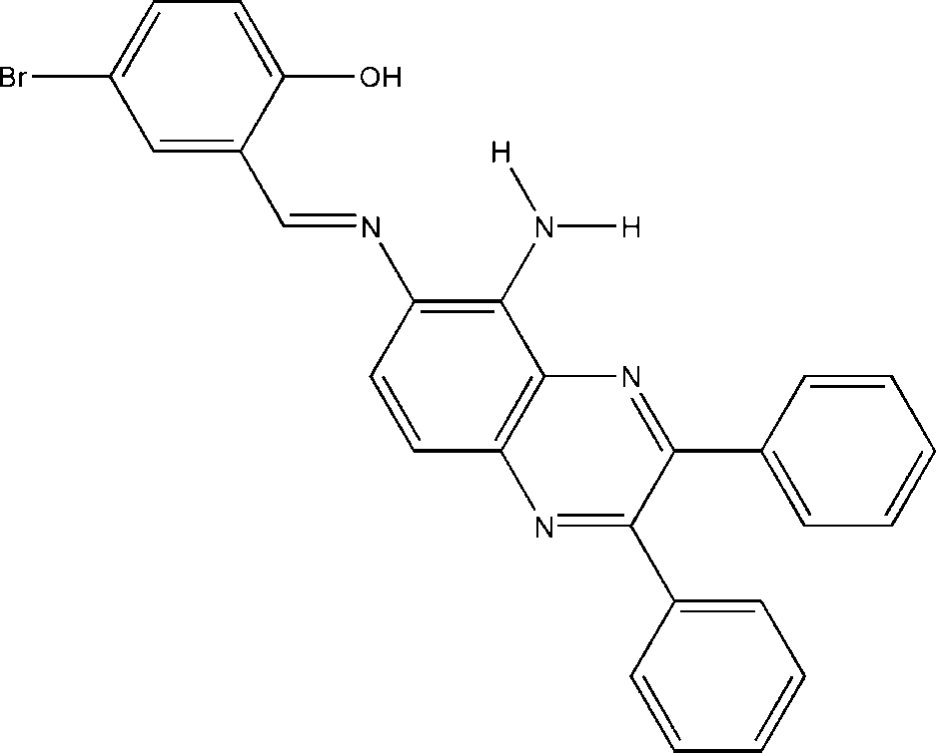


## Experimental

### 

#### Crystal data

C_27_H_19_BrN_4_O
*M* *_r_* = 495.37
Monoclinic, 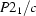

*a* = 22.923 (5) Å
*b* = 7.344 (5) Å
*c* = 12.573 (5) Å
β = 92.070 (5)°
*V* = 2115.2 (17) Å^3^
*Z* = 4
Mo *K*α radiation
μ = 1.97 mm^−1^
*T* = 100.0 (1) K
0.39 × 0.37 × 0.08 mm

#### Data collection

Bruker SMART APEXII CCD area-detector diffractometer
Absorption correction: multi-scan (*SADABS*; Bruker, 2005 ▶) *T*_min_ = 0.510, *T*_max_ = 0.853
22554 measured reflections
6205 independent reflections
3722 reflections with *I* > 2σ(*I*)
*R*_int_ = 0.070

#### Refinement

*R*[*F* ^2^ > 2σ(*F* ^2^)] = 0.045
*wR*(*F* ^2^) = 0.142
*S* = 1.06
6205 reflections
305 parameters
H atoms treated by a mixture of independent and constrained refinement
Δρ_max_ = 0.61 e Å^−3^
Δρ_min_ = −1.07 e Å^−3^

### 

Data collection: *APEX2* (Bruker, 2005 ▶); cell refinement: *APEX2*; data reduction: *SAINT* (Bruker, 2005 ▶); program(s) used to solve structure: *SHELXTL* (Sheldrick, 2008 ▶); program(s) used to refine structure: *SHELXTL*; molecular graphics: *SHELXTL*; software used to prepare material for publication: *SHELXTL* and *PLATON* (Spek, 2003 ▶).

## Supplementary Material

Crystal structure: contains datablocks global, I. DOI: 10.1107/S1600536808017716/sj2516sup1.cif

Structure factors: contains datablocks I. DOI: 10.1107/S1600536808017716/sj2516Isup2.hkl

Additional supplementary materials:  crystallographic information; 3D view; checkCIF report

## Figures and Tables

**Table 1:** Hydrogen-bond geometry (Å, °)

*D*—H⋯*A*	*D*—H	H⋯*A*	*D*⋯*A*	*D*—H⋯*A*
O1—H1*O*1⋯N1	0.83	1.83	2.586 (4)	151
N2—H2*N*2⋯N4	0.88 (4)	2.35 (4)	2.740 (4)	107 (3)
N2—H1*N*2⋯N1	0.88 (4)	2.44 (4)	2.756 (4)	102 (3)

**Fig. 1.**
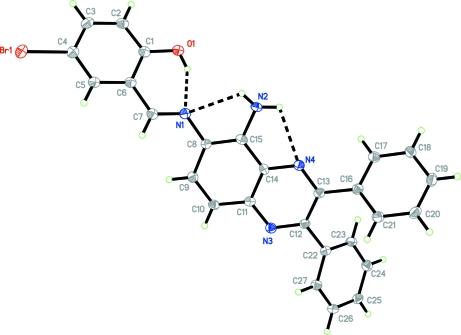
The molecular structure of the title compound, showing 50% probability displacement ellipsoids and the atomic numbering. Intramolecular interactions are drawn as dashed lines.

**Fig. 2.**
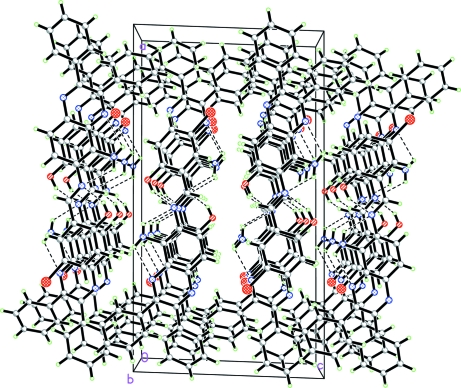
The crystal packing of (I), viewed along the b-axis with hydrogen bonds drawn as dashed lines.

## supplementary crystallographic information

### Comment

Schiff bases are among the most prevalent mixed-donor ligands in coordination chemistry. Schiff bases and their biologically active complexes have been studied over several decades (Anderson *et al.,* 1997; Blower 1998; Corden *et al.,* 1996; Govindasamy *et al.,* 1999; Granovski *et al.,* 1993; Li & Chang, 1991; Shahrokhian *et al.*, 2000). 2-hydroxy Schiff base ligands are of interest mainly due to the existence of O—H···N and O···H—N type hydrogen bonds and tautomerization between the phenol-imine and keto-amine forms (Unaleroglu & Hokelek, 2002; Kia *et al.*, 2004). This type of tautomerism plays an important role for distinguishing their photochromic and thermochromic properties (Cohen & Schmidt, 1964). Knowing the structures of free Schiff bases in solution and in the solid state is important in view of the intramolecular hydrogen bonding and comparison with the structure of Schiff base complexes. In view of the importance of these organic ligands, the title compound (I) was synthesized and its crystal structure is reported here.

In the title compound (Fig. 1), intramolecular O—H···N, and N—H···N hydrogen bonds form six and five-membered rings, producing S(6) and S(5) ring motifs, respectively (Bernstein *et al.*, 1995). The two phenyl substituents on the quinoxaline unit are inclined at an angle of 17.87 (17)° to one another. They also form dihedral angles of 38.96 (15) and 44.46 (15)° with the ten–membered quinoxaline ring. In the crystal packing (Fig. 2), molecules are stacked along the *b* axis by π···π interactions with *Cg2*···*Cg3* distances ranging from 3.628 (3) – 3.729 (3) Å: symmetry codes 1 - *x*, 1/2 + *y*, 3/2 - *z* and 1 - *x*, -1/2 + *y*, 3/2 - *z*; *Cg2* and *Cg3* are the centroids of the C1–C6 and C8/C9/C10/C11/C14/C15 phenyl rings, respectively. The crystal structure is stabilized by intramolecular O—H···N, and N—H···N hydrogen bonds, weak intermolecular N—H···O and C—H···O hydrogen bonds, and π···π interactions.

### Experimental

A mixture of *o*-diaminoquinoxaline (313 mg, 1 mmol) and 5-bromo salicylaldehyde (210 mg, 1 mmol) was suspended in 30 ml absolute ethanol. The reaction mixture was stirred under reflux for 1 h. After cooling, the precipitate was filtered, washed with ethanol and ether and dried under vacuum. Single crystals suitable for *X*-ray diffraction were obtained by evaporation of a mixed chloroform-ethanol (3/1, *v*/*v*) solution at room temperature.

### Refinement

The H-atoms attached to O1 were located from the difference Fourier map and refined as riding with the parent atom with an isotropic thermal parameter 1.2 times that of the parent atom. The H-atoms bound to N were located in a difference Fourier map and refined freely with the parent atom with an isotropic thermal parameter 1.2 times that of the parent atom. The rest of the hydrogen atoms were positioned geometrically [C—H = 0.93 Å] and refined using a riding model, with thermal parameters 1.2 times those of the parent atoms.

### Crystal data

**Table:**

C_27_H_19_BrN_4_O	*F*_000_ = 1008
*M**_r_* = 495.37	*D*_x_ = 1.556 Mg m^−^^3^
Monoclinic, *P*2_1_/*c*	Mo *K*α radiation λ = 0.71069 Å
Hall symbol: -P 2ybc	Cell parameters from 4509 reflections
*a* = 22.923 (5) Å	θ = 2.9–28.3º
*b* = 7.344 (5) Å	µ = 1.97 mm^−^^1^
*c* = 12.573 (5) Å	*T* = 100.0 (1) K
β = 92.070 (5)º	Block, yellow
*V* = 2115.2 (17) Å^3^	0.39 × 0.37 × 0.08 mm
*Z* = 4	

### Data collection

**Table:**

Bruker SMART APEXII CCD area-detector diffractometer	6205 independent reflections
Radiation source: fine-focus sealed tube	3722 reflections with *I* > 2σ(*I*)
Monochromator: graphite	*R*_int_ = 0.070
*T* = 100.0(1) K	θ_max_ = 30.1º
φ and ω scans	θ_min_ = 0.9º
Absorption correction: multi-scan(SADABS; Bruker, 2005)	*h* = −32→32
*T*_min_ = 0.510, *T*_max_ = 0.853	*k* = −10→8
22554 measured reflections	*l* = −16→17

### Refinement

**Table:**

Refinement on *F*^2^	Secondary atom site location: difference Fourier map
Least-squares matrix: full	Hydrogen site location: inferred from neighbouring sites
*R*[*F*^2^ > 2σ(*F*^2^)] = 0.045	H atoms treated by a mixture of independent and constrained refinement
*wR*(*F*^2^) = 0.142	*w* = 1/[σ^2^(*F*_o_^2^) + (0.0698*P*)^2^] where *P* = (*F*_o_^2^ + 2*F*_c_^2^)/3
*S* = 1.07	(Δ/σ)_max_ = 0.001
6205 reflections	Δρ_max_ = 0.62 e Å^−^^3^
305 parameters	Δρ_min_ = −1.07 e Å^−^^3^
Primary atom site location: structure-invariant direct methods	Extinction correction: none

### Special details

**Table:**

Experimental. The low-temperature data was collected with the Oxford Cyrosystem Cobra low-temperature attachment.
Geometry. All e.s.d.'s (except the e.s.d. in the dihedral angle between two l.s. planes) are estimated using the full covariance matrix. The cell e.s.d.'s are taken into account individually in the estimation of e.s.d.'s in distances, angles and torsion angles; correlations between e.s.d.'s in cell parameters are only used when they are defined by crystal symmetry. An approximate (isotropic) treatment of cell e.s.d.'s is used for estimating e.s.d.'s involving l.s. planes.
Refinement. Refinement of *F*^2^ against ALL reflections. The weighted *R*-factor *wR* and goodness of fit *S* are based on *F*^2^, conventional *R*-factors *R* are based on *F*, with *F* set to zero for negative *F*^2^. The threshold expression of *F*^2^ > σ(*F*^2^) is used only for calculating *R*-factors(gt) *etc*. and is not relevant to the choice of reflections for refinement. *R*-factors based on *F*^2^ are statistically about twice as large as those based on *F*, and *R*- factors based on ALL data will be even larger.

### Fractional atomic coordinates and isotropic or equivalent isotropic displacement parameters (Å^2^)

**Table:**

	*x*	*y*	*z*	*U*_iso_*/*U*_eq_	
Br1	0.259515 (13)	0.53134 (5)	0.59180 (3)	0.02170 (12)	
N1	0.52495 (11)	0.6363 (4)	0.7764 (2)	0.0171 (6)	
N2	0.60814 (13)	0.5339 (4)	0.9285 (2)	0.0186 (6)	
N4	0.72399 (11)	0.5294 (4)	0.8819 (2)	0.0154 (6)	
N3	0.76064 (11)	0.6677 (4)	0.6873 (2)	0.0169 (6)	
O1	0.44969 (9)	0.7345 (3)	0.91363 (18)	0.0206 (5)	
H1O1	0.4813	0.7101	0.8877	0.025*	
C1	0.40804 (13)	0.6934 (4)	0.8389 (3)	0.0177 (7)	
C2	0.34965 (14)	0.7191 (4)	0.8608 (3)	0.0188 (7)	
H2	0.3398	0.7694	0.9256	0.023*	
C3	0.30643 (13)	0.6715 (5)	0.7882 (3)	0.0205 (8)	
H3	0.2675	0.6883	0.8044	0.025*	
C4	0.32022 (13)	0.5985 (5)	0.6910 (3)	0.0183 (7)	
C5	0.37775 (13)	0.5769 (4)	0.6645 (3)	0.0176 (7)	
H5	0.3866	0.5305	0.5982	0.021*	
C6	0.42273 (13)	0.6246 (4)	0.7373 (3)	0.0162 (7)	
C7	0.48319 (13)	0.6048 (5)	0.7087 (3)	0.0166 (7)	
H7	0.4917	0.5688	0.6400	0.010 (8)*	
C8	0.58402 (12)	0.6335 (4)	0.7490 (3)	0.0146 (7)	
C9	0.60282 (13)	0.6891 (4)	0.6473 (3)	0.0174 (7)	
H9	0.5752	0.7203	0.5945	0.021*	
C10	0.66057 (13)	0.6973 (4)	0.6258 (3)	0.0170 (7)	
H10	0.6721	0.7344	0.5591	0.020*	
C11	0.70293 (13)	0.6494 (4)	0.7053 (3)	0.0159 (7)	
C14	0.68498 (13)	0.5891 (4)	0.8062 (3)	0.0135 (6)	
C15	0.62412 (13)	0.5842 (4)	0.8283 (3)	0.0157 (7)	
C12	0.79874 (13)	0.6218 (5)	0.7641 (3)	0.0165 (7)	
C13	0.78033 (13)	0.5390 (4)	0.8603 (3)	0.0161 (7)	
C16	0.82122 (13)	0.4561 (5)	0.9406 (3)	0.0174 (7)	
C17	0.81113 (14)	0.4750 (5)	1.0488 (3)	0.0185 (7)	
H17	0.7793	0.5428	1.0702	0.022*	
C18	0.84782 (13)	0.3944 (5)	1.1246 (3)	0.0199 (7)	
H18	0.8408	0.4086	1.1965	0.024*	
C19	0.89531 (14)	0.2918 (5)	1.0935 (3)	0.0223 (8)	
H19	0.9205	0.2393	1.1444	0.027*	
C20	0.90483 (14)	0.2686 (5)	0.9858 (3)	0.0224 (8)	
H20	0.9358	0.1970	0.9645	0.027*	
C21	0.86863 (13)	0.3509 (5)	0.9104 (3)	0.0196 (7)	
H21	0.8758	0.3364	0.8386	0.024*	
C22	0.86036 (13)	0.6776 (4)	0.7467 (3)	0.0166 (7)	
C23	0.89337 (13)	0.7640 (5)	0.8263 (3)	0.0198 (7)	
H23	0.8786	0.7758	0.8939	0.024*	
C24	0.94824 (14)	0.8330 (5)	0.8060 (3)	0.0220 (8)	
H24	0.9701	0.8916	0.8595	0.026*	
C25	0.97015 (14)	0.8138 (5)	0.7048 (3)	0.0215 (8)	
H25	1.0068	0.8603	0.6906	0.026*	
C26	0.93829 (14)	0.7271 (5)	0.6262 (3)	0.0215 (8)	
H26	0.9535	0.7137	0.5590	0.026*	
C27	0.88320 (13)	0.6588 (5)	0.6463 (3)	0.0198 (7)	
H27	0.8615	0.6004	0.5925	0.024*	
H2N2	0.6362 (17)	0.487 (5)	0.970 (3)	0.024*	
H1N2	0.5740 (17)	0.479 (5)	0.925 (3)	0.024*	

### Atomic displacement parameters (Å^2^)

**Table:**

	*U*^11^	*U*^22^	*U*^33^	*U*^12^	*U*^13^	*U*^23^
Br1	0.01741 (16)	0.02067 (18)	0.0269 (2)	−0.00067 (13)	−0.00060 (13)	0.00133 (17)
N1	0.0177 (13)	0.0150 (15)	0.0187 (16)	0.0014 (10)	0.0023 (11)	0.0015 (12)
N2	0.0185 (13)	0.0240 (15)	0.0134 (15)	−0.0013 (12)	0.0037 (11)	0.0011 (13)
N4	0.0168 (12)	0.0141 (13)	0.0152 (15)	−0.0001 (10)	0.0007 (10)	−0.0019 (12)
N3	0.0178 (13)	0.0166 (15)	0.0163 (15)	−0.0007 (10)	0.0011 (11)	−0.0018 (12)
O1	0.0200 (11)	0.0251 (14)	0.0169 (13)	−0.0002 (9)	0.0033 (10)	−0.0002 (11)
C1	0.0213 (16)	0.0129 (17)	0.0190 (19)	−0.0012 (12)	0.0019 (14)	0.0034 (14)
C2	0.0228 (16)	0.0151 (17)	0.0189 (19)	0.0015 (12)	0.0044 (14)	0.0051 (14)
C3	0.0166 (15)	0.0174 (18)	0.028 (2)	0.0021 (12)	0.0043 (14)	0.0079 (15)
C4	0.0191 (15)	0.0133 (16)	0.022 (2)	−0.0001 (12)	0.0016 (13)	0.0046 (15)
C5	0.0197 (15)	0.0155 (17)	0.0178 (19)	0.0018 (12)	0.0028 (13)	0.0011 (13)
C6	0.0186 (15)	0.0131 (17)	0.0168 (18)	−0.0003 (12)	0.0026 (13)	0.0018 (14)
C7	0.0198 (15)	0.0130 (16)	0.0171 (19)	0.0009 (12)	0.0010 (13)	0.0022 (14)
C8	0.0155 (14)	0.0128 (16)	0.0156 (18)	−0.0012 (11)	0.0012 (12)	−0.0013 (13)
C9	0.0194 (15)	0.0153 (17)	0.0174 (19)	−0.0003 (12)	0.0001 (13)	0.0022 (14)
C10	0.0190 (15)	0.0168 (17)	0.0155 (18)	−0.0007 (12)	0.0027 (13)	0.0025 (14)
C11	0.0179 (15)	0.0135 (16)	0.0163 (18)	−0.0003 (11)	0.0034 (13)	−0.0002 (13)
C14	0.0181 (15)	0.0085 (14)	0.0137 (17)	0.0002 (11)	−0.0012 (12)	−0.0002 (13)
C15	0.0216 (15)	0.0112 (15)	0.0145 (18)	−0.0016 (12)	0.0027 (13)	−0.0036 (13)
C12	0.0154 (14)	0.0182 (17)	0.0161 (18)	0.0002 (12)	0.0028 (13)	−0.0020 (14)
C13	0.0164 (14)	0.0151 (16)	0.0169 (18)	0.0011 (12)	0.0021 (12)	−0.0046 (15)
C16	0.0155 (14)	0.0176 (17)	0.0192 (19)	−0.0021 (12)	0.0018 (12)	0.0008 (15)
C17	0.0195 (15)	0.0181 (16)	0.0180 (19)	−0.0017 (13)	0.0024 (13)	−0.0013 (15)
C18	0.0201 (16)	0.0218 (18)	0.0179 (19)	−0.0010 (13)	0.0003 (13)	0.0030 (15)
C19	0.0174 (16)	0.025 (2)	0.024 (2)	0.0000 (13)	−0.0026 (14)	0.0043 (16)
C20	0.0173 (15)	0.0227 (19)	0.028 (2)	0.0054 (13)	0.0036 (14)	0.0021 (16)
C21	0.0201 (16)	0.0191 (18)	0.0198 (19)	0.0015 (12)	0.0037 (14)	0.0013 (15)
C22	0.0160 (14)	0.0162 (17)	0.0180 (18)	0.0006 (12)	0.0034 (13)	−0.0004 (14)
C23	0.0188 (15)	0.0250 (19)	0.0158 (19)	−0.0009 (13)	0.0034 (13)	−0.0012 (15)
C24	0.0202 (16)	0.0253 (19)	0.020 (2)	−0.0019 (13)	0.0001 (14)	0.0012 (16)
C25	0.0162 (15)	0.027 (2)	0.022 (2)	0.0006 (13)	0.0034 (14)	0.0050 (16)
C26	0.0203 (16)	0.028 (2)	0.0165 (19)	0.0026 (13)	0.0049 (14)	0.0002 (16)
C27	0.0210 (16)	0.0221 (19)	0.0164 (19)	0.0029 (13)	0.0015 (13)	−0.0019 (15)

### Geometric parameters (Å, °)

**Table:**

Br1—C4	1.900 (4)	C10—H10	0.9300
N1—C7	1.279 (4)	C11—C14	1.418 (4)
N1—C8	1.409 (3)	C14—C15	1.433 (4)
N2—C15	1.376 (4)	C12—C13	1.431 (4)
N2—H2N2	0.88 (4)	C12—C22	1.495 (4)
N2—H1N2	0.88 (4)	C13—C16	1.483 (5)
N4—C13	1.331 (4)	C16—C17	1.396 (5)
N4—C14	1.355 (4)	C16—C21	1.397 (4)
N3—C12	1.322 (4)	C17—C18	1.382 (5)
N3—C11	1.357 (4)	C17—H17	0.9300
O1—C1	1.350 (4)	C18—C19	1.391 (4)
O1—H1O1	0.8254	C18—H18	0.9300
C1—C2	1.389 (4)	C19—C20	1.390 (5)
C1—C6	1.425 (5)	C19—H19	0.9300
C2—C3	1.368 (5)	C20—C21	1.377 (5)
C2—H2	0.9300	C20—H20	0.9300
C3—C4	1.382 (5)	C21—H21	0.9300
C3—H3	0.9300	C22—C23	1.386 (5)
C4—C5	1.381 (4)	C22—C27	1.391 (4)
C5—C6	1.398 (5)	C23—C24	1.388 (4)
C5—H5	0.9300	C23—H23	0.9300
C6—C7	1.452 (4)	C24—C25	1.392 (5)
C7—H7	0.9300	C24—H24	0.9300
C8—C15	1.380 (5)	C25—C26	1.365 (5)
C8—C9	1.424 (4)	C25—H25	0.9300
C9—C10	1.362 (4)	C26—C27	1.390 (4)
C9—H9	0.9300	C26—H26	0.9300
C10—C11	1.413 (5)	C27—H27	0.9300
			
C7—N1—C8	122.5 (3)	N2—C15—C14	118.5 (3)
C15—N2—H2N2	116 (3)	C8—C15—C14	118.8 (3)
C15—N2—H1N2	110 (3)	N3—C12—C13	121.2 (3)
H2N2—N2—H1N2	118 (4)	N3—C12—C22	115.2 (3)
C13—N4—C14	117.4 (3)	C13—C12—C22	123.3 (3)
C12—N3—C11	118.3 (3)	N4—C13—C12	120.8 (3)
C1—O1—H1O1	106.6	N4—C13—C16	115.7 (3)
O1—C1—C2	119.6 (3)	C12—C13—C16	123.4 (3)
O1—C1—C6	121.3 (3)	C17—C16—C21	118.5 (3)
C2—C1—C6	119.1 (3)	C17—C16—C13	120.0 (3)
C3—C2—C1	120.9 (3)	C21—C16—C13	121.4 (3)
C3—C2—H2	119.6	C18—C17—C16	120.8 (3)
C1—C2—H2	119.6	C18—C17—H17	119.6
C2—C3—C4	120.4 (3)	C16—C17—H17	119.6
C2—C3—H3	119.8	C17—C18—C19	120.0 (3)
C4—C3—H3	119.8	C17—C18—H18	120.0
C5—C4—C3	120.6 (3)	C19—C18—H18	120.0
C5—C4—Br1	119.7 (3)	C20—C19—C18	119.5 (3)
C3—C4—Br1	119.7 (2)	C20—C19—H19	120.2
C4—C5—C6	120.1 (3)	C18—C19—H19	120.2
C4—C5—H5	119.9	C21—C20—C19	120.3 (3)
C6—C5—H5	119.9	C21—C20—H20	119.8
C5—C6—C1	118.9 (3)	C19—C20—H20	119.8
C5—C6—C7	120.0 (3)	C20—C21—C16	120.8 (3)
C1—C6—C7	121.1 (3)	C20—C21—H21	119.6
N1—C7—C6	121.0 (3)	C16—C21—H21	119.6
N1—C7—H7	119.5	C23—C22—C27	119.1 (3)
C6—C7—H7	119.5	C23—C22—C12	121.0 (3)
C15—C8—N1	116.6 (3)	C27—C22—C12	119.6 (3)
C15—C8—C9	120.6 (3)	C22—C23—C24	120.6 (3)
N1—C8—C9	122.7 (3)	C22—C23—H23	119.7
C10—C9—C8	121.2 (3)	C24—C23—H23	119.7
C10—C9—H9	119.4	C23—C24—C25	119.3 (3)
C8—C9—H9	119.4	C23—C24—H24	120.3
C9—C10—C11	119.8 (3)	C25—C24—H24	120.3
C9—C10—H10	120.1	C26—C25—C24	120.5 (3)
C11—C10—H10	120.1	C26—C25—H25	119.7
N3—C11—C10	120.4 (3)	C24—C25—H25	119.7
N3—C11—C14	119.8 (3)	C25—C26—C27	120.2 (3)
C10—C11—C14	119.8 (3)	C25—C26—H26	119.9
N4—C14—C11	121.6 (3)	C27—C26—H26	119.9
N4—C14—C15	118.6 (3)	C26—C27—C22	120.2 (3)
C11—C14—C15	119.8 (3)	C26—C27—H27	119.9
N2—C15—C8	122.7 (3)	C22—C27—H27	119.9
			
O1—C1—C2—C3	−177.3 (3)	C11—C14—C15—N2	−176.8 (3)
C6—C1—C2—C3	3.1 (5)	N4—C14—C15—C8	−175.8 (3)
C1—C2—C3—C4	−0.7 (5)	C11—C14—C15—C8	2.0 (5)
C2—C3—C4—C5	−1.7 (5)	C11—N3—C12—C13	5.7 (5)
C2—C3—C4—Br1	179.5 (2)	C11—N3—C12—C22	−169.0 (3)
C3—C4—C5—C6	1.6 (5)	C14—N4—C13—C12	4.5 (4)
Br1—C4—C5—C6	−179.5 (2)	C14—N4—C13—C16	−174.3 (3)
C4—C5—C6—C1	0.7 (5)	N3—C12—C13—N4	−9.6 (5)
C4—C5—C6—C7	−178.9 (3)	C22—C12—C13—N4	164.7 (3)
O1—C1—C6—C5	177.3 (3)	N3—C12—C13—C16	169.1 (3)
C2—C1—C6—C5	−3.1 (5)	C22—C12—C13—C16	−16.6 (5)
O1—C1—C6—C7	−3.1 (5)	N4—C13—C16—C17	−39.1 (4)
C2—C1—C6—C7	176.6 (3)	C12—C13—C16—C17	142.2 (3)
C8—N1—C7—C6	−174.5 (3)	N4—C13—C16—C21	137.8 (3)
C5—C6—C7—N1	−175.3 (3)	C12—C13—C16—C21	−40.9 (5)
C1—C6—C7—N1	5.0 (5)	C21—C16—C17—C18	1.2 (5)
C7—N1—C8—C15	−150.5 (3)	C13—C16—C17—C18	178.1 (3)
C7—N1—C8—C9	33.4 (5)	C16—C17—C18—C19	−0.4 (5)
C15—C8—C9—C10	−0.8 (5)	C17—C18—C19—C20	−1.2 (5)
N1—C8—C9—C10	175.2 (3)	C18—C19—C20—C21	2.0 (5)
C8—C9—C10—C11	0.3 (5)	C19—C20—C21—C16	−1.2 (5)
C12—N3—C11—C10	179.8 (3)	C17—C16—C21—C20	−0.4 (5)
C12—N3—C11—C14	2.5 (4)	C13—C16—C21—C20	−177.3 (3)
C9—C10—C11—N3	−175.9 (3)	N3—C12—C22—C23	132.1 (3)
C9—C10—C11—C14	1.4 (5)	C13—C12—C22—C23	−42.5 (5)
C13—N4—C14—C11	3.7 (5)	N3—C12—C22—C27	−41.7 (4)
C13—N4—C14—C15	−178.6 (3)	C13—C12—C22—C27	143.7 (3)
N3—C11—C14—N4	−7.5 (5)	C27—C22—C23—C24	0.8 (5)
C10—C11—C14—N4	175.2 (3)	C12—C22—C23—C24	−173.0 (3)
N3—C11—C14—C15	174.8 (3)	C22—C23—C24—C25	−0.4 (5)
C10—C11—C14—C15	−2.5 (5)	C23—C24—C25—C26	−0.4 (5)
N1—C8—C15—N2	2.1 (5)	C24—C25—C26—C27	0.7 (5)
C9—C8—C15—N2	178.4 (3)	C25—C26—C27—C22	−0.3 (5)
N1—C8—C15—C14	−176.6 (3)	C23—C22—C27—C26	−0.4 (5)
C9—C8—C15—C14	−0.3 (5)	C12—C22—C27—C26	173.4 (3)
N4—C14—C15—N2	5.5 (4)		

### Hydrogen-bond geometry (Å, °)

**Table:**

*D*—H···*A*	*D*—H	H···*A*	*D*···*A*	*D*—H···*A*
O1—H1O1···N1	0.83	1.83	2.586 (4)	151
N2—H2N2···N4	0.88 (4)	2.35 (4)	2.740 (4)	107 (3)
N2—H1N2···N1	0.88 (4)	2.44 (4)	2.756 (4)	102 (3)
